# Effect of Reaction Parameters on the Synthesis of Cyclodextrin-Based Nanostructured Polymers for Drug Delivery

**DOI:** 10.3390/polym17060709

**Published:** 2025-03-07

**Authors:** Sema Salgın, Hasan Hüseyin Eke, Nagihan Soyer, Uğur Salgın

**Affiliations:** 1 Department of Chemical Engineering, Faculty of Engineering, Sivas Cumhuriyet University, 58140 Sivas, Turkey; nsoyer@cumhuriyet.edu.tr (N.S.); usalgin@cumhuriyet.edu.tr (U.S.); 2 Farma-Tek Pharmaceutical Industry and Trade Inc., 39100 Kırklareli, Turkey; hasaneke90@gmail.com

**Keywords:** cyclodextrin, epichlorohydrin, nanostructure, drug carrier

## Abstract

In this study, cyclodextrin-based nanostructures (CDNSs) were synthesized through the cross-linking of cyclodextrin (CD) with epichlorohydrin (ECH) as a cross-linker. Two types of CDNSs, α-CDNS and β-CDNS, were prepared to systematically investigate the influence of reaction parameters—such as the solubilization time of α-CD and β-CD, the molar ratio of ECH to CD, and NaOH concentration—on the physicochemical properties of the final product. Naproxen (NAP), a poorly water-soluble drug, was selected as a model compound to assess the drug-loading capacity of the synthesized CDNSs. The effect of each reaction parameter on NAP integration into the CDNSs was examined at varying weight ratios. The optimal reaction conditions were determined to be a solubilization time of 6 h, an ECH/CD molar ratio of 8/1, and an NaOH concentration of 33%. Under these conditions, the NAP loading efficiency of α-CDNSs was calculated as 67.12%. Comparative analysis revealed that α-CDNSs outperformed β-CDNSs in terms of drug-loading capacity. Additionally, the synthesized CDNSs and NAP-loaded CDNSs were characterized using FTIR, DSC, XRD, SEM, and Zetasizer analyses, while the NAP concentration was determined by HPLC.

## 1. Introduction

Cyclodextrins (CDs) are natural cyclic oligosaccharides derived from the enzymatic degradation of starch, which have gained prominence in pharmaceutical sciences due to their unique structure [1]. The naturally occurring CDs—α-, β-, and γ-CDs—consist of six, seven, and eight glucopyranose units, respectively. Since these glucose units adopt a chair conformation, CDs assume a truncated cone shape that is crucial to their functionality [2]. The glucose subunits are linked by α-1,4-glucosidic bonds, forming a relatively hydrophobic inner cavity and a hydrophilic outer surface decorated with numerous hydroxyl groups [3]. This unique architecture enables CDs to form host–guest inclusion complexes with hydrophobic molecules, including organic compounds and drugs, making them ideal candidates for various supramolecular applications [4,5]. CDs can enhance solubility and stability, reduce volatility, mask undesirable flavors, and control the release of guest molecules. In pharmaceutical applications, they improve drug bioavailability, stability, solubility, and dissolution rate [6].

Despite their advantages, conventional cyclodextrin–drug complexes face several limitations, such as restricted stability, suboptimal solubility, and reduced therapeutic efficacy, which hinder their broader application [7]. For instance, the low drug-loading capacity and uncontrolled release kinetics limit their effectiveness in drug delivery. To address these challenges, cyclodextrin-based nanostructures, or nanosponges (CDNSs), have emerged as transformative drug delivery systems. These nanostructures are hyper-branched supramolecular polymers with a three-dimensional network [8,9,10]. Notably, CDNSs exhibit a remarkable capacity for forming both inclusion and non-inclusion complexes with a wide range of compounds, including hydrophobic, hydrophilic, and charged molecules [11,12]. This adaptability allows CDNSs to enhance the solubilization of poorly water-soluble drugs, increase drug-loading capacity, improve bioavailability through prolonged release, and protect against environmental and enzymatic degradation [13,14].

The synthesis of CDNSs utilizes the abundant hydroxyl groups on the hydrophilic outer surface of CDs as reactive sites for covalent bonding. The hydroxyl groups exhibit different reactivities depending on their position: the 6-OH groups on the upper rim are the most accessible and frequently modified, the 2-OH groups on the lower rim are the most acidic due to hydrogen bonding, and the 3-OH groups, also on the lower rim, are sterically hindered and thus less reactive under standard conditions [15].

**Scheme 1:**
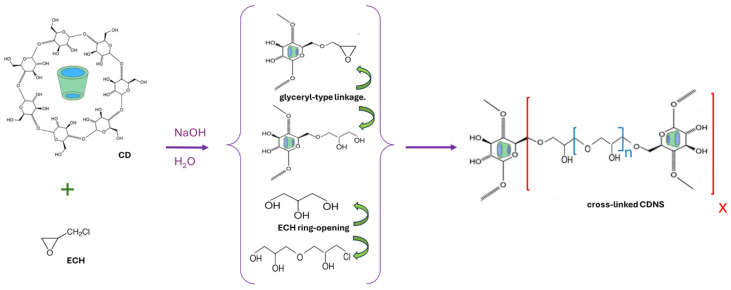
Schematic representation of the polycondensation reaction between CD and ECH under basic conditions.

CDNSs are synthesized through cross-linking, a process involving highly reactive cross-linkers with at least two active sites capable of forming covalent bonds with the hydroxyl groups of CDs. This cross-linking occurs via condensation polymerization, requiring the activation of CD hydroxyl groups by the electron-withdrawing groups of the cross-linker. Once activated, these hydroxyl groups are attacked by the nucleophilic sites of the cross-linker, which typically binds to the primary hydroxyl group at the 6-OH position [7]. Various cross-linkers, such as epichlorohydrin (ECH) [16], pyromellitic dianhydride [4,17], hexamethylenediisocyanate [18], carbonyldiimidazole [19], and diphenyl carbonate [13,20], have been employed for the synthesis of CDNSs. Among these, ECH is the most commonly used cross-linker in polysaccharide chemistry because of its high reactivity. ECH contains both an epoxide group and a chloroalkyl moiety, which react with the hydroxyl groups of CDs in an alkaline medium. The polycondensation reaction between ECH and CD produces a heterogeneous mixture of CD glyceryl ethers. Depending on the reaction conditions, particularly the degree of cross-linking, ECH materials can form cross-linked polymers that are either water-soluble or water-insoluble. In a basic medium, the reactive cross-linking agent can form bonds with cyclodextrin molecules (the cross-linking step) and/or with itself (the polymerization step), resulting in multiple interconnected cyclodextrin rings and the formation of a three-dimensional polymer network. Scheme 1 illustrates this reaction, showing the nucleophilic attack of CD hydroxyl groups on the epoxide ring of ECH under basic conditions, leading to the formation of glyceryl-type linkages and a cross-linked CDNS network [21,22,23].

Several studies highlight the potential of ECH-cross-linked CD-based nanostructures for improving drug solubility, dissolution, and bioavailability. For instance, Mura et al. (2002) co-ground naproxen (NAP) with water-soluble and water-insoluble CD–ECH polymers, achieving significant reductions in NAP crystallinity and markedly enhanced dissolution. The insoluble polymer exerted a stronger amorphizing effect, whereas the soluble polymer yielded greater dissolution improvements in simple physical mixtures due to its higher solubility. Notably, a 10:90 (*w*/*w*) drug-to-carrier ratio resulted in complete amorphization and a more than 30-fold increase in dissolution efficiency relative to untreated NAP [24]. Nie et al. (2011) employed a newly modified, water-soluble β-CD–ECH polymer to enhance the dissolution rate and oral bioavailability of glipizide. Co-evaporated complexes formed a 1:1 ratio with the drug and outperformed HP-β-CD in terms of solubility and dissolution. In vivo tests in beagle dogs confirmed a marked increase in glipizide bioavailability [25]. Similarly, Jug et al. (2011) reported that a water-soluble ECH–CD polymer exhibited a stronger affinity for triclosan than native CD, producing fully amorphous complexes with superior dissolution and antimicrobial activity against *Streptococcus mutans*. While hydrophilic polymers improved solubility in phase solubility studies, they did not promote solid-state interactions as effectively as ECH–CD [26].

A major challenge in CDNS synthesis is ensuring reproducibility and optimizing the reaction conditions to achieve the desired physicochemical properties. The selection of the cross-linking agent and reaction parameters significantly impact the final material properties. Among various cross-linkers, ECH is widely used due to its high reactivity under basic conditions and its mechanism is the best known. The presence of NaOH plays a crucial role in activating CD hydroxyl groups, thereby influencing the cross-linking and polymerization steps. To this end, critical reaction parameters, such as the solubilization time of CDs, the type of CDs (α-CD and β-CD), the molar ratio of ECH to CDs, and the base concentration (NaOH), were systematically investigated. Furthermore, the study investigates the loading of NAP into CDNSs at varying weight ratios to optimize drug-loading performance. NAP was chosen due to its poor water solubility and widespread use as a nonsteroidal anti-inflammatory drug, making it an ideal candidate to evaluate the efficiency of CDNSs as drug carriers. This research study aims to provide a robust understanding of the parameters influencing CDNS synthesis, contributing to the development of more efficient drug delivery systems.

## 2. Materials and Methods

### 2.1. Materials

α-cyclodextrin, β-cyclodextrin, S-Naproxen and epichlorohydrin (1-chloro-2,3-epoxypropane) were provided by Sigma-Aldrich Chemie GmbH (Taufkirchen, Germany). The solvents used in HPLC analyses were liquid chromatography grade (Merck KGaA, Darmstadt, Germany). All other chemicals and reagents were obtained from Merck KGaA (Darmstadt, Germany). Milli-Q water (Millipore Co., Billerica, MA, USA) was used throughout the experiments.

### 2.2. Synthesis of α- and β-CDNSs

α-CDNS and β-CDNS are prepared by reacting dried α-CD and β-CD with epichlorohydrin (ECH) in a basic medium. Each species of CD was dissolved individually in a NaOH solution with concentrations of 25%, 33%, and 40% (*w*/*w*) while being mechanically stirred (300 rpm) at a temperature of 30 °C for predetermined solubilization times of 3, 6, and 8 h. At the end of each dissolution period, the solution was vigorously stirred (600 rpm) for 3 h at 50 °C after rapidly adding the desired amount of the cross-linker ECH (an ECH/CD molar ratio of 6/1, 8/1, and 10/1). The polymerization reaction was stopped after 3 h by adding acetone. After decantation, acetone was removed, and the solution was kept at 50 °C overnight. After cooling, the solution was neutralized with 6 N HCl. The obtained solution was evaporated and precipitated by adding anhydrous ethanol. The solution was decanted, and the resulting white CDNSs were dried at 50 °C for 2 h [27,28].

### 2.3. Preparation of NAP-CDNS Complexes

NAP was dispersed in 20 mL aqueous suspensions of different types of CDNS at weight ratios of 1/4, 1/6, and 1/8 (NAP to CDNS) and stirred at 200 rpm at 25 °C for 24 h. After 24 h, the suspensions were centrifuged at 7000 rpm for 15 min to separate the uncomplexed drug. Samples from the supernatant were analyzed by HPLC to determine the NAP concentration. The remaining samples were oven-dried at 50 °C and used for further analysis. NAP loading efficiency (NLE) was calculated using the following equation (Equation (1)):

(1) $$NLE,\%=\frac{C_{NS}}{C_{O}}\times 100$$

where C_O_ is the initial concentration of NAP in aqueous suspensions, C_NS_ is the concentration of NAP in CDNSs after loading. The C_NS_ was calculated using a mass balance. NAP concentration was constant (25 mg/mL) in all drug-loading processes.

The loading content (LC%) of NAP–CDNS was determined by measuring the concentration of unencapsulated NAP using UV–vis spectroscopy (UV-1800, Shimadzu, Kyoto, Japan) at 254 nm. A known amount of the NAP–CDNS complex was dispersed in 2-propanol and sonicated for 10 min to disrupt the complex. The loading content (LC%) was then calculated according to the following equation (Equation (2)) [29].

(2) $$LC\%=\frac{Weight\;of\;NAP\;in\;CDNSs}{Weigh\;of\;NAP\;loaded\;CDNSs}\times 100$$

### 2.4. HPLC Analyses

The concentrations of NAP were measured with HPLC (Dionex UltiMate 3000, Thermo Fisher Scientific, Beaverton, OR, USA) by using a CHIRALPAK AD-H column (Diacel, Osaka, Japan) at 25 °C. In the analyses, *n*-hexane (90%):2-propanol (10%) was used as the mobile phase at a flow rate of 1 mL/min, and UV detection was performed at a wavelength of 254 nm.

### 2.5. Characterizations of CDNSs

Structural characterizations of CDNSs before and after NAP loading experiments were obtained by FTIR spectroscopy (Spectrum 100, Perkin Elmer, Norwalk, CT, USA) for different reaction conditions. The FTIR spectra were recorded at room temperature with a resolution of 4 cm^−1^ over the range of 400–4000 cm^−1^. Thermograms of NAP, CD and CDNSs were obtained using a DSC (DSC 7020, Hitachi, Tokyo, Japan) instrument. The powdered samples (1.5 mg) were placed into an aluminum sample pan and sealed. Thermograms were run from 10 to 450 °C using a temperature gradient of 10 °C/min with a sealed empty pan as a reference sample. Pure nitrogen (99.9%) was used as the carrier gas at 20 mL/min.

The average hydrodynamic diameter (Z_ave._), polydispersity index (PDI), and zeta potential (ZP) values of CDNSs were determined using dynamic light scattering (DLS) and phase analysis light scattering (PALS), respectively, with a Zetasizer (NanoZS, Malvern Panalytical, Worcestershire, UK) equipped with a He-Ne laser (633 nm) and operating at a scattering angle of 173°. Each measurement was performed three times, and the table presents the mean values of three independent measurements. The morphologies of the CDNSs were determined by SEM (LEO 440, Zeiss, Jena, Germany) analysis. The XRD patterns of the samples were obtained by XRD (D8 Advance, Bruker AXS, Karlsruhe, Germany) using Cu K_α_ radiation.

## 3. Results and Discussion

### 3.1. Effect of CD Solubilization Time on the CDNS Synthesis

Natural CDs exhibit limited aqueous solubility. The pKa of the secondary hydroxyl groups is 12.3 ± 0.2, while the primary hydroxyl groups have a pKa ranging from 15 to 16. The hydroxyl groups attached to the rim begin to deprotonate at a pH of approximately 12. Consequently, strong bases can be utilized to deprotonate and solubilize CDs [30]. In our experiments, NaOH was used to dissolve a significant amount of CD in an aqueous solution, forming alcoholate sites [28,31]. These hydroxyl groups can then react with one reactive group of the bifunctional agent, ECH.

The α-CD and β-CD were added separately to a medium containing a 33% (*w*/*w*) NaOH solution. The mixture was then allowed to solubilize for 3, 6, and 8 h at 30 °C and 300 rpm. Subsequently, ECH was added to the reaction medium at a molar ratio of 8/1 (ECH/CD). The procedure described in Section 2.2 was then followed.

The average hydrodynamic diameter (Z_ave._), polydispersity index (PDI), and zeta potential (ZP) values of α-CDNS and β-CDNS drug carrier systems are presented in Table 1. The abbreviations 3hα-CDNS, 6hα-CDNS, and 8hα-CDNS represent α-CDNS synthesized after 3, 6, and 8 h of solubilization time of α-CD, respectively. Similar abbreviations were used for different solubilization times of β-CD.

The Z_ave._ values for α-CDNSs and β-CDNSs ranged from 254 nm to 321 nm. It can be observed that the Z_ave._ values of α- and β-CDs are similar for the same solubilization times. An increase in Z_ave._ was observed when the solubilization time was increased from 3 h to 6 h, while no significant change was observed in Z_ave._ after 8 h of solubilization time for both CDNS types. The PDI values of α-CDNS and β-CDNS synthesized at varying solubilization times ranged from 0.36 to 0.45, indicating the size distribution range in the formulations [32]. These values suggest that the synthesized CDNS particles exhibit mid-range polydispersity [33].

ZP, an indicator of surface charge, reflects the ionization of polar groups on a surface or the adsorption of ions from a solution onto the surface. Moreover, ZP is a key parameter in colloidal systems, as it determines the stability of the system by indicating whether aggregation occurs. Colloidal systems exhibit better stability when the ZP value is approximately −30 mV or +30 mV [32,34]. Since the ZP values of α- and β-CDNS are close to this threshold, it can be concluded that the CDNSs exhibit sufficient colloidal stability. The CDNSs showed a negative ZP, possibly due to the presence of unreacted hydroxyl groups that remained after the reaction with ECH, as well as chloride ions. A high ZP creates repulsive forces between CDNSs, preventing aggregation [35]. Extending the solubilization time from 3 h to 6 h increased the absolute value of the surface charge of the CDNSs. However, a further extension of the time resulted in a slight decrease in the absolute value of the ZP.

**Figure 1:**
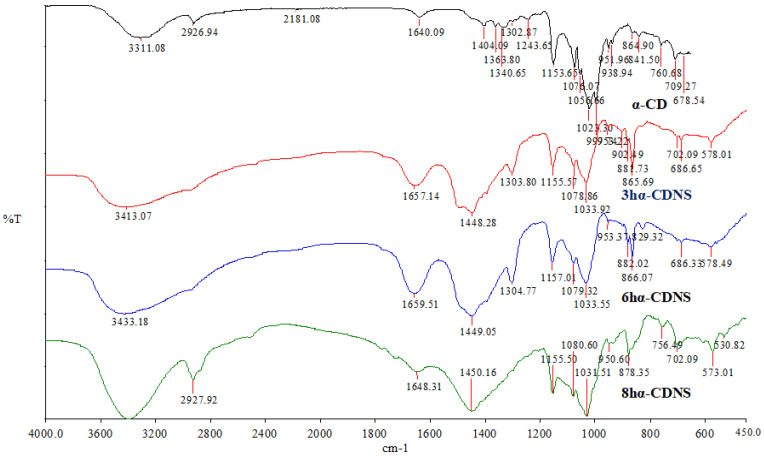
FTIR spectra of CDNSs synthesized at different solubilization times of α-CD.

**Figure 2:**
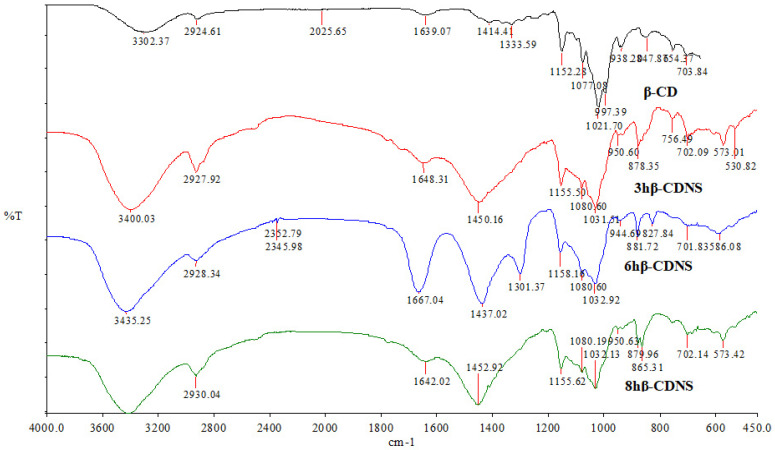
FTIR spectra of CDNSs synthesized at different solubilization times of β-CD.

The FTIR spectra of α- CDs (Figure 1) and β-CDs (Figure 2) exhibited characteristic peaks at 3311 cm^−1^ and 3302 cm^−1^ (the O-H stretching vibration), 2925 and 2927 cm^−1^ (the C-H stretching vibration), ~1414 and 1334 cm^−1^ (the C-H deformation vibration), ~1154 cm^−1^ (the C-C stretching vibration), and ~1022 cm^−1^ (the C-O-C stretching vibration). In addition, a peak at 1640 cm^−1^, corresponding to the deformation bands of H-O-H, indicates the presence of water in the CDs [36,37]. In the FTIR spectra of α-CDNSs (Figure 1) and β-CDNSs (Figure 2) prepared at different solubilization times, the O-H stretching vibration at approximately 3400 cm^−1^, the C-H stretching vibration at 2927–2930 cm^−^^1^, and the bending vibrations at 1437–1453 cm^−1^, as well as the C-O-C stretching vibration at 1031–1033 cm^−^^1^ due to the normal alkane structure, were observed. The similarities between the spectra of the original CDs and CDNSs indicate that the basic structural units are preserved in CDNSs. However, some bands are shifted or broadened, confirming that a cross-linking reaction occurs between CDs and ECH components, resulting in the formation of new chemical bonds [11,13].

The increased intensity of the O-H band (~3400 cm^−^^1^) suggests the formation of new O-H groups during the cross-linking reaction. The intensities of the C-H (2920 and 1450 cm^−^^1^) and C-O-C (1032 cm^−^^1^) bands also increased, further indicating the formation of cross-linked CDNSs. The stretching vibration of C-O-C at 1032 cm^−^^1^ provides evidence of the cross-linking process. Furthermore, the increased intensity of the C-H vibration at 1450 cm^−^^1^ is attributed to the introduction of glyceryl bridges during the reaction [36].

**Figure 3:**
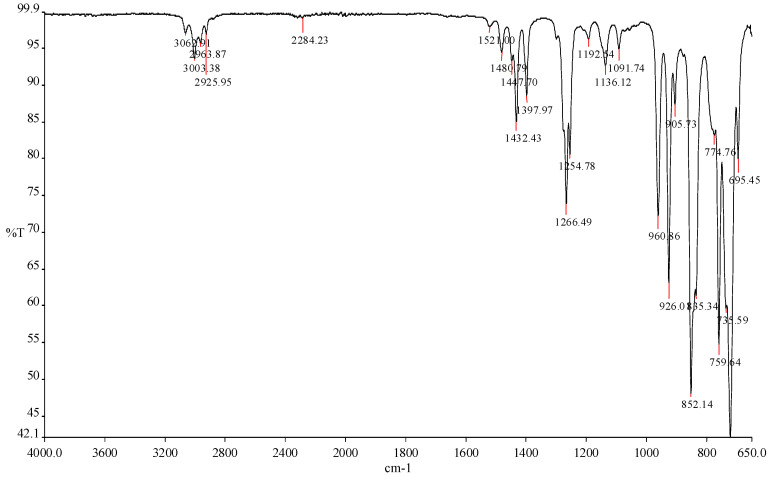
FTIR spectrum of ECH.

No characteristic peaks corresponding to ECH, such as the CH_2_Cl rocking band at 1266 cm^−^^1^ and the CH_2_Cl wagging band at 1254 cm^−^^1^ (Figure 3), were observed in the synthesized CDNSs [27]. This indicates that ECH reacted almost completely with CDs.

### 3.2. NAP-CDNS Complexes: Impact of Solubilization Time on Complex Formation and Their Characterization

NAP was incorporated into the CDNS by continuous stirring at 25 °C and 200 rpm for 24 h at different NAP/CDNS weight ratios (1/4, 1/6, and 1/8 *w*/*w*). NAP concentrations in the samples taken at the beginning and end of the loading process were analyzed using HPLC. In all loading experiments, the initial NAP concentration was consistently maintained at 25 mg/mL. NAP loading efficiency (NLE, %) was calculated using Equation (1). The results, including Z_ave._, PDI, ZP, and NLE, are summarized in Table 2. The Z_ave._ of the original CDNSs ranged from 253 to 321 nm (Table 1), whereas that of the NAP-loaded CDNSs increased to 317–665 nm (Table 2). This increase in Z_ave._ values suggests the successful formation of complexes between the model drug NAP and the CDNS drug delivery system. Similarly, Swaminathan et al. (2010) reported an increase in the size of camptothecin-loaded β-CDNSs [38]. Likewise, studies by Alwattar and Mehanna (2024) [39] and Dhakar et al. (2019) [40] indicated that the particle size of blank nanosponges was slightly smaller than that of active-loaded nanosponges, which is consistent with the observations of this study. A slight increase in PDI values was observed in NAP-loaded CDNSs compared to their unloaded counterparts.

In all studied formulations, the ZP values of NAP-loaded CDNS slightly decreased in absolute terms compared to the blank CDNS. This decrease is attributed to the blocking of -OH groups by the drug [20]. Additionally, there was a slight increase in the PDI values of CDNSs after drug loading.

By increasing the solubilization time from 3 h to 6 h, the NLE improved for both types of CDNSs. However, when the solubilization time was extended to 8 h, the loading efficiency of CDNSs decreased across all loading ratios. This decline may be attributed to the excessive deprotonation of CD hydroxyl groups in NaOH, generating alkoxide ions that readily undergo S_N_2 reactions with ECH, leading to structural modifications [41]. Additionally, prolonged solubilization could result in excessive cross-linking, reducing the porosity and accessibility of the CDNS matrix for drug encapsulation. These findings indicate that 6 h of solubilization in NaOH is the optimal duration for achieving high NAP loading efficiency. At this optimal solubilization time, α-CDNSs exhibited a higher NLE, approximately 28% greater than that of β-CDNSs under all loading conditions. This difference can be attributed to the higher solubility of α-CD compared to β-CD [42].

In Table 2, when the NAP/CDNS loading ratio was changed from 1/4 to 1/6, the naproxen loading efficiency (NLE)—also referred to as encapsulation efficiency (EE) in the literature—generally increased, whereas at 1/8, it decreased in all formulations. Although increasing the CDNS amount theoretically provides more nanocavities for NAP encapsulation, our experimental data show that the highest loading efficiency occurred at an intermediate ratio (1/6 NAP/α-CDNS). Specifically, the NLE values for NAP/CDNS at 1/4, 1/6, and 1/8 were 51.80%, 67.12%, and 45.96%, respectively, while the corresponding NAP loading content (LC) values were 8.62%, 11.04%, and 7.67%.

For comparison, Salem et al. (2023) synthesized various CDNS systems using different cross-linkers—carbonyldiimidazole (CDI), pyromellitic dianhydride (PMDA), and citric acid (CA)—to encapsulate Budesonide (BUD). BUD was loaded at different weight ratios (1/1, 1/2, 1/3, 1/4) using three methods (A, B, and C), and the resulting BUD–CDNS systems were assessed for encapsulation EE% and LC%. In β-CD:CDI nanosponge formulations, EE generally increased up to an intermediate ratio (e.g., 1/3) before declining, while LC tended to decrease as the loading ratio rose. In β-CD:PMDA systems, raising the loading ratio from 1/1 to 1/4 under Methods B and C improved EE, though LC remained nearly unchanged under Method B and decreased under Method C. Meanwhile, β-CD:CA-based nanosponges showed higher EE values up to a 1/3 loading ratio under both Methods B and C, followed by a drop. Regarding LC, a rise was observed up to 1/3 under Method B, then declined, whereas under Method C it generally decreased with higher loading ratios [43]. Allahyari et al. (2021) synthesized CDNSs using β-CD and CDI as a cross-linker in molar ratios of 1:2 and 1:4. They subsequently loaded the antiandrogen drug flutamide (FLT) into this carrier system at a 1:5 (*w*/*w*) FLT/CDNS ratio. For the CDNS prepared at a β-CD/CDI ratio of 1:2, the encapsulation efficiency and loading capacity were 36.05% and 6%, respectively. In contrast, for the CDNS synthesized at a β-CD/CDI ratio of 1:4, these values increased to 56.5% and 9.4% [29]. Hoti et al. (2023) employed citric acid as a cross-linker to produce both molecularly imprinted nanosponges (MIP-NSs) and non-imprinted nanosponges (NIP-NSs) for melatonin encapsulation. The highest LC of 6.51% and an EE of 39.11% were recorded in the NIP-NS. Overall, NIP-NSs (LC: 4–7%) displayed a greater loading capacity than MIP-NSs (LC: 1–1.5%), whereas MIP-NSs exhibited higher EE values (60–90%) compared to NIP-NSs (20–40%) [44].

These studies indicate that parameters such as the type of cross-linker, the CD/cross-linker molar ratio, the drug-loading method, and the drug/CDNS loading ratio all exert significant influences on both the encapsulation efficiency and loading capacity of CDNS. Overall, these findings underscore that, much like in our study, there is often an optimal ratio for maximizing encapsulation and loading capacity.

**Figure 4:**
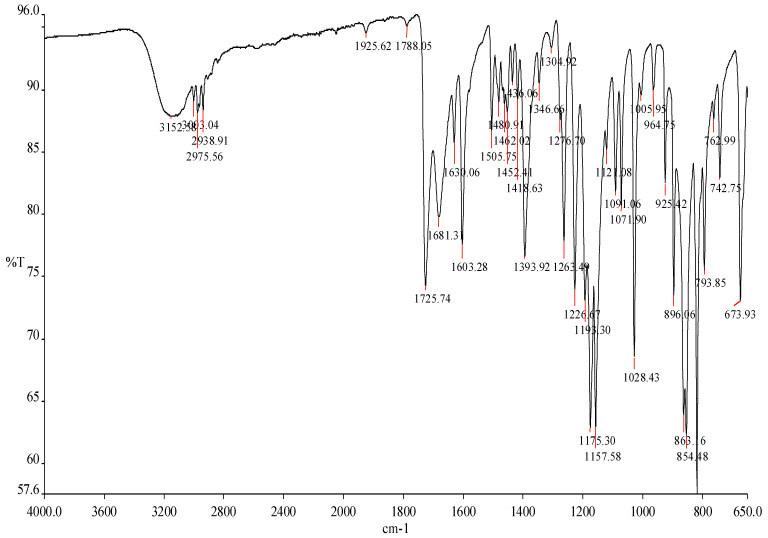
FTIR spectrum of NAP.

**Figure 5:**
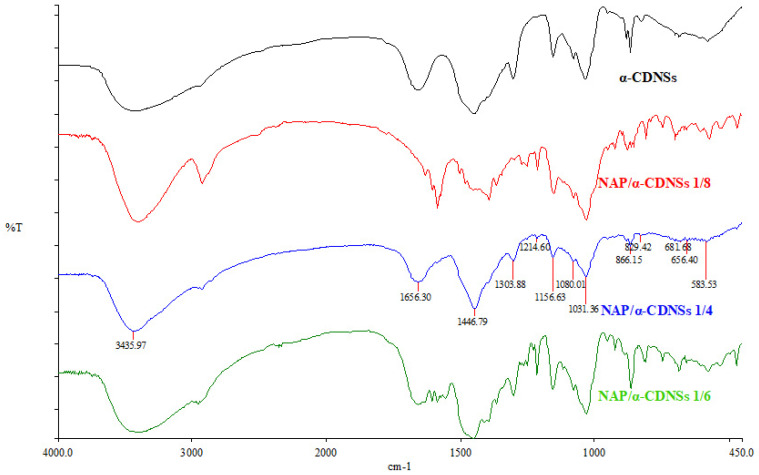
Comparison of FTIR spectra of α-CDNSs and NAP-loaded α-CDNSs at different loading ratios.

**Figure 6:**
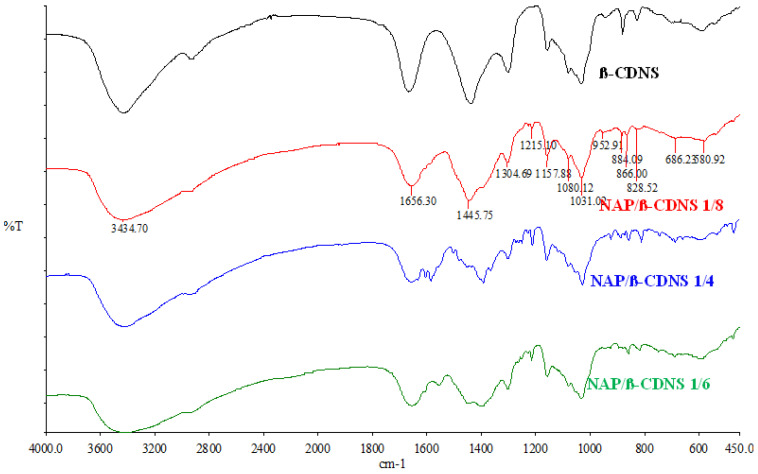
Comparison of FTIR spectra of β-CDNSs and NAP-loaded β-CDNSs at different loading ratios.

The FTIR spectra of NAP, NAP-loaded α-CDNS, and NAP-loaded β-CDNS are shown in Figure 4, Figure 5 and Figure 6, respectively. The characteristic absorption bands observed in the IR spectrum of the model drug NAP correspond to C-O stretching at 1028 cm^−1^, and 1226 cm^−1^ corresponds to -O- stretching, CH_3_ bending at 1393 cm^−1^, while the absorption bands at 1681 cm^−1^ and 1725 cm^−1^ correspond to anti-symmetric and symmetric C-O stretching vibrations (Figure 4) [45].

After complexation, the NAP absorption peak at 1725 cm^−^^1^ was no longer observed in the FTIR spectra of both CDNS carriers (Figure 4 and Figure 5). Most NAP bands disappeared in the complex spectrum, indicating that NAP was encapsulated into the host cavity [46]. Additionally, the characteristic NAP peak at 1226 cm^−^^1^ shifted slightly to lower wavenumbers—1214 cm^−^^1^ for α-CDNS and 1215 cm^−^^1^ for β-CDNS—indicating the formation of host–guest interactions between NAP and CDNS [47]. This shift, along with the potential broadening of the carbonyl band, suggests the entrapment of NAP within the nanosponge pores and the formation of inclusion complexes. These spectral changes are consistent with previously reported evidence of host–guest interactions upon cyclodextrin complexation [48,49]. During this process, no covalent bonds are formed or broken, and the guest molecule remains in rapid equilibrium between the complexed and free states in solution [50]. The formation of the complex is driven by several factors, including the release of enthalpy-rich water molecules from the cyclodextrin cavity, electrostatic interactions, hydrogen bonding, the release of conformational strain, and charge transfer interactions [51].

After determining the optimal solubilization time of CDs in NaOH as 6 h, the synthesized CDNSs and drug-loaded CDNSs (prepared at the optimal NAP/CDNS ratio of 1/6) were characterized using DSC, XRD, and SEM analyses to evaluate the formation of inclusion complexes and their potential as drug delivery systems. DSC analysis is widely utilized to confirm findings from spectroscopic analyses [52] and is considered the most commonly used thermal technique for investigating solid-state interactions between drugs and CDs. This method provides valuable insights into their physical and energetic properties [53].

**Figure 7:**
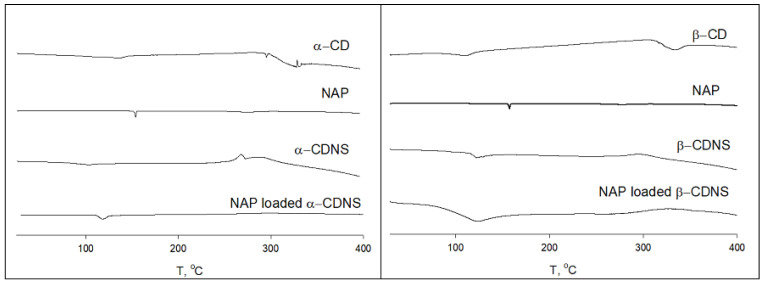
DSC thermograms of CDs, NAP, CDNSs, and NAP-loaded CDNSs.

The DSC thermograms of CDs, CDNSs, NAP, and NAP-loaded CDNSs are shown in Figure 7. Both α-CD and β-CD exhibited endothermic peaks at approximately 140 °C and 110 °C, respectively, attributed to the loss of water from the solid state prior to thermal degradation. Additionally, the crystalline α-CD and β-CD showed a characteristic decomposition peak around 330 °C. In contrast, this peak was absent in the thermograms of CDNSs, suggesting an alteration in their thermal stability.

In the DSC curves of α-CDNS and β-CDNS, endothermic peaks were observed at approximately 104 °C and 120 °C, respectively, which correspond to the expulsion of water molecules from the cyclodextrin CD cavities [54,55]. The DSC curve of NAP exhibited a sharp endothermic peak at approximately 153 °C, corresponding to its melting point [24]. This peak was suppressed in the DSC thermograms of NAP-loaded CDNSs, confirming the inclusion of NAP within the cyclodextrin cavities. Matencio et al. (2024) stated that the almost complete disappearance of the endothermic transition of a drug is a strong indication of inclusion complex formation [37]. The suppression of this peak also suggests the loss of NAP’s crystalline nature due to its molecular dispersion within the cross-linked polymer matrix [54]. This phenomenon is commonly interpreted as evidence of true inclusion complex formation. Conversely, the presence of a residual melting peak—whether broadened or shifted to a lower temperature—indicates the presence of free drug molecules in the crystalline state, suggesting incomplete or absent inclusion complexation [53].

**Figure 8:**
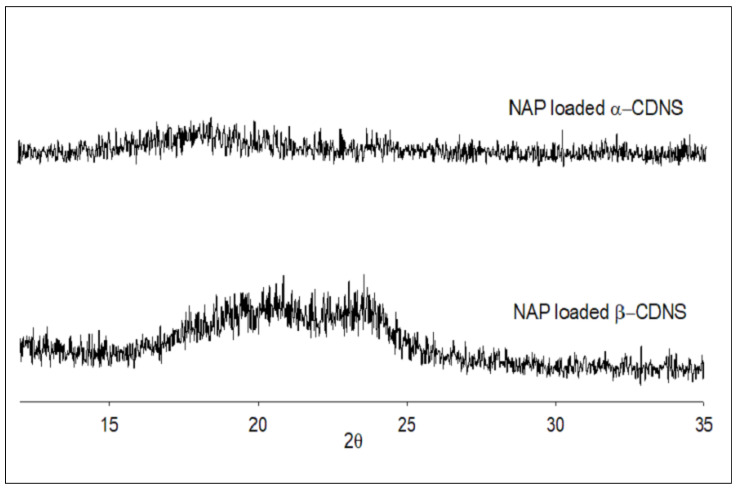
XRD patterns of NAP-loaded α-CDNSs and β-CDNSs.

In the DSC thermograms of NAP-loaded α-CDNSs and β-CDNSs, shifted and broadened endothermic peaks were observed at 118 °C and 124 °C, respectively. Therefore, XRD analyses of NAP-loaded CDNSs were performed to further verify the phenomena observed in the DSC analysis (Figure 8).

Literature-reported XRD diffractograms of NAP [24], α-CD [56], and β-CD [4] display multiple diffraction peaks, confirming their crystalline nature. In contrast, the XRD patterns of NAP-loaded CDNS drug carriers present a single broad band, with the characteristic peaks of both the drug and excipient disappearing (Figure 8). This transformation indicates that NAP is no longer in its crystalline form but is instead incorporated into the polymeric matrix in a disordered crystalline phase, an amorphous state, or a solid-state dissolved form, supporting the formation of an inclusion complex between NAP and CDNSs. The formation of the inclusion complex between NAP and CDNS—facilitated by hydrogen bonding and van der Waals forces—not only disrupts NAP’s crystalline structure, transforming it into an amorphous form, but also enhances its solubility and provides a more controlled drug release profile. This amorphous state is characterized by a disordered structure and higher free energy compared to its crystalline counterpart, significantly reducing the energy barrier for dissolution. Consequently, this thermodynamic driving force results in higher apparent water solubility and an increased dissolution rate. Moreover, the effective drug–polymer interactions promote a more homogeneous dispersion of NAP within the polymer matrix, further enhancing its solubility and bioavailability. Typically, crystalline drugs suffer from solubility and release limitations; however, the complexation process overcomes these issues by converting the drug into a readily diffusible amorphous state, thereby controlling its release. Overall, the reduction in crystallinity serves as an additional factor contributing to the enhanced solubility of NAP in the CDNS [57,58,59].

**Figure 9:**
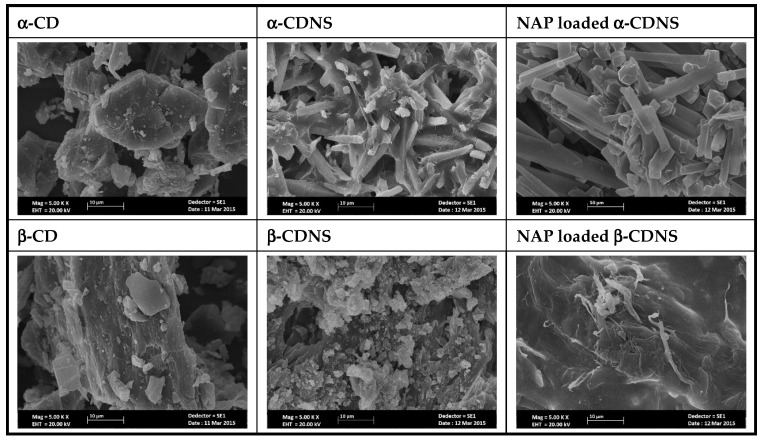
SEM images of CDs, CDNSs and NAP-loaded CDNSs.

Figure 9 presents the SEM images illustrating the surface morphology of α- and β-CDs, α- and β-CDNSs, and NAP-loaded α- and β-CDNSs. The α- and β-CDs exhibited irregularly shaped, crystalline structures, while both blank and NAP-loaded CDNSs appeared as rough, porous particles with an amorphous texture. These morphological transformations indicate successful cross-linking within the polymeric network, leading to the formation of a three-dimensional porous structure [60]. Notably, blank CDNSs displayed visible nanocavities on their surfaces, which were subsequently filled upon NAP loading, further supporting the formation of an inclusion complex. The disappearance of well-defined crystalline surfaces in the NAP-loaded CDNSs suggests the amorphization of the drug within the polymer matrix. These findings align with previous observations by Gupta et al. (2021) [61] and Pant and Bhattacharya (2024) [62], who reported similar morphological changes upon drug loading in cyclodextrin-based nanosponges.

### 3.3. Effect of Molar Ratio of ECH/α-CD

One of the key reaction engineering parameters in cyclodextrin polymer synthesis is the concentration of the cross-linker ECH, which contains two reactive functional groups capable of forming bonds with CD molecules and/or with itself. The end polymers are mixtures comprising CD units interconnected by repeating glyceryl linkers. Adjusting the ratio of CD to ECH is essential for tailoring the polymer’s loading capacity and optimizing its release profile [28,63,64].

In this part of the study, the effect of the cross-linker ECH on CDNS synthesis was examined using α-CD as the reactant, as α-CDNSs demonstrated the highest NAP loading efficiency. The experiments were conducted with a dissolution time of 6 h in a 33% NaOH solution, employing ECH/α-CD molar ratios of 6/1, 8/1, and 10/1. The ZP, Z_ave._, and PDI values of the synthesized α-CDNS drug carrier systems are summarized in Table 3.

The Z_ave._ value was observed to decrease as the ECH/α-CD molar ratio increased. At a 6/1 molar ratio, the amount of the cross-linker appeared insufficient to achieve complete CDNS formation, resulting in larger and less uniform particles. This observation is consistent with Swaminathan et al. (2010), who reported that lower cross-linker amounts lead to the reduced cross-linking of β-CD, thereby decreasing the availability of active sites for complexation [38]. Conversely, at a 10/1 molar ratio, the increased ECH concentration likely accelerates the reaction and enhances the degree of cross-linking, producing smaller particle sizes. These findings align with Renard et al. (1997), who demonstrated that higher cross-linker concentrations form denser cross-linked networks, leading to smaller particles [28]. Similarly, studies on cyclodextrin-based polymers have indicated that the cross-linker-to-cyclodextrin molar ratio plays a critical role in determining particle size and cross-linking density [64]. In agreement with these findings, Gholibegloo et al. (2019) reported that a higher ECH/β-CD molar ratio results in a smaller hydrodynamic size, which could be attributed to the lower swelling ratio of CDNS at higher ECH/β-CD molar ratios [65].

The most pronounced increase in Z_ave._ was observed in α-CDNSs synthesized at an ECH/α-CD molar ratio of 8/1, which also exhibited the highest drug-loading efficiency (Table 4). This is likely due to the optimal cross-linking density at this ratio, where intermediate cross-linker concentrations were found to provide an ideal balance between network flexibility and drug encapsulation capacity. The ZP values ranged from −25 mV to −34 mV, indicating that the CDNSs exhibited a stable colloidal suspension. The PDI values of the α-CDNS synthesized at different ECH/α-CD molar ratios ranged from 0.409 to 0.546, indicating a medium level of polydispersity.

The CDNS drug carrier systems, synthesized at varying ECH/CD molar ratios, were loaded with NAP at different loading ratios, and their properties—such as particle size and surface charge—were analyzed (Table 4). Following NAP loading, the Z_ave._ values increased across all systems compared to the original drug carriers. This observation is consistent with the concept that guest molecule loading, such as drug encapsulation, can expand polymer structures due to interactions between the polymeric network and the drug molecules. Similarly, Salem et al. (2023) reported a significant increase in Z_ave._ between blank and Budesonide-βCD-NS systems, confirming the successful loading of Budesonide into the nanosponge [43].

At a 6/1 ECH/αCD molar ratio, NLE ranges from 20.80% to 25.56%, whereas at an 8/1 ratio, NLE increases significantly, reaching 51.80–67.12%. However, at a 10/1 ratio, NLE decreases again to 22.20–26.40%. This trend suggests that a moderate cross-linker ratio (8/1) provides the highest loading efficiency, while both lower (6/1) and excessively high (10/1) ratios may negatively impact NLE. At a 6/1 ratio, insufficient cross-linking may result in a less stable network with inadequate cavities for encapsulation, leading to lower efficiency. Conversely, the decrease at 10/1 could be attributed to excessive cross-linking, which may create a more rigid structure with reduced accessibility for guest molecules.

### 3.4. Effect of NaOH Concentration

The effect of NaOH concentration on α-CDNS synthesis was investigated at 25%, 33%, and 40% (*w*/*w*). The other reaction conditions were set to the optimal parameters that yielded the highest NAP loading efficiency, namely a 6 h solubilization time of α-CD in NaOH and an ECH/α-CD molar ratio of 8/1. The characteristic properties of the α-CDNS synthesized under these conditions are presented in Table 5.

Z_ave._ increased with NaOH concentration from 25% to 33% but decreased at 40%, indicating a possible structural shift in the polymer network. The PDI values ranged between 0.409 and 0.479, indicating moderate polydispersity.

As demonstrated in Table 6, the process of NAP loading onto α-CDNS, which was synthesized at varying concentrations of NaOH, resulted in a modest decline in the absolute ZP values in comparison to the blank CDNS. This observation indicates an alteration in the surface charge distribution. Additionally, increases in Z_ave._ and PDI values were noted, confirming the successful loading of NAP onto the carrier system. The highest NAP loading efficiency was achieved at a NaOH concentration of 33%, likely due to an optimal balance between polymer flexibility and cross-linking density, whereas the lowest was observed at 25%, where reduced deprotonation may have limited efficient cross-linking.

Jiang et al. (2012) reported that NaOH concentrations above 30% are required for the production of water-insoluble CD polymers [22]. They stated that increasing NaOH concentration promotes the deprotonation of –OH groups at the C-2 or C-3 positions in the glucopyranose structure of CDs, thereby facilitating their reaction with ECH. Similarly, Renard et al. (1997) observed that polycondensation does not occur when NaOH concentration exceeds 33%, and that at 50%, NaOH accelerates the precipitation of CDs. They also suggested that lower NaOH concentrations favor ECH-CD polycondensation [28]. Therefore, at a high NaOH concentration (40%), the increased cross-linking density likely resulted in a more compact and rigid polymer network, leading to reduced particle size and decreased NAP loading efficiency. Considering both the experimental and literature findings, 33% NaOH was determined as the optimal concentration for synthesizing water-soluble CDNS, providing a balance between polymerization, solubility, and drug-loading efficiency.

As shown in Table 6, NLE values strongly depend on NaOH concentration and the NAP/α-CDNS ratio, highlighting the role of cross-linking density in drug encapsulation. The highest loading efficiency (67.12%) was observed at 33% NaOH with a 1/6 NAP/α-CDNS ratio, suggesting that this condition provides an optimal balance between polymer structure and drug encapsulation capacity. In contrast, the lowest loading efficiency (7.44%) was recorded at 25% NaOH with a 1/4 NAP/α-CDNS ratio, suggesting that insufficient cross-linking at lower NaOH concentrations limits the polymer network’s ability to encapsulate NAP efficiently. The decrease in NLE at 40% NaOH suggests that excessive cross-linking may lead to a more compact structure, limiting the available sites for drug loading. These findings support previous studies [22,28] indicating that moderate NaOH concentrations promote the formation of well-structured, water-soluble CDNS, thereby maximizing drug-loading efficiency.

## 4. Conclusions

In this study, the effects of critical reaction parameters—including the solubilization time of α-CD and β-CD), the molar ratio of ECH to CDs, and the NaOH concentration—on CDNS-based drug carrier synthesis were investigated. These parameters were optimized to achieve the best performance in NAP loading at different ratios, ultimately identifying the most effective conditions. The optimal reaction conditions were determined to be a solubilization time of 6 h, an ECH/CD molar ratio of 8/1, and an NaOH concentration of 33%. Under these conditions, the NAP loading efficiency of α-CDNSs was calculated as 67.12% for the 1/6 NAP/α-CDNS loading ratio. The findings highlight that α-CDNSs serve as a more efficient drug carrier for NAP encapsulation compared to β-CDNSs due to their favorable structural and physicochemical properties. FTIR analyses confirmed the successful synthesis of CDNSs and the effective integration of NAP into their structure. Additionally, DSC, SEM and XRD analyses indicated that NAP was incorporated into the CDNSs in an amorphous state, which can enhance drug solubility and bioavailability. Zeta potential measurements further verified the stability of the synthesized CDNSs and the successful encapsulation of the guest molecule. Overall, this study provides a comprehensive understanding of the factors influencing CDNS synthesis and drug encapsulation efficiency. The optimized α-CDNS formulations may present a promising strategy for enhancing the solubility of poorly water-soluble drugs, highlighting their potential for drug delivery applications.

## Figures and Tables

**Table 1:** Z_ave._, ZP, and PDI values of CDNSs synthesized from α- and β-CDs at different solubilization times.

	3hα-CDNS	3hβ-CDNS	6hα-CDNS	6hβ-CDNS	8hα-CDNS	8hβ-CDNS
Z_ave._(nm)	253.90	268.60	313.10	321.00	309.60	311.20
PDI	0.357	0.422	0.409	0.434	0.445	0.451
ZP (mV)	−23.20	−24.80	−33.60	−28.70	−25.50	−24.20

**Table 2:** The effect of solubilization time on the Z_ave._, ZP, PDI and NLE values of α-CDNS and β-CDNS loaded with NAP.

	NAP/3hα-CDNS			NAP/3hβ-CDNS			NAP/6hα-CDNS			NAP/6hβ-CDNS			NAP/8hα-CDNS			NAP/8hβ-CDNS		
NAP/CDNS (*w*/*w*)	1/4	1/6	1/8	1/4	1/6	1/8	1/4	1/6	1/8	1/4	1/6	1/8	1/4	1/6	1/8	1/4	1/6	1/8
Z_ave_. (nm)	402.10	525.40	557.10	413.10	407.20	395.70	656.00	665.30	620.20	410.90	562.90	530.70	490.10	370.90	450.30	410.20	317.20	319.30
PDI	0.573	0.420	0.559	0.442	0.523	0.521	0.537	0.418	0.575	0.450	0.628	0.704	0.514	0.476	0.514	0.462	0.630	0.845
ZP (mV)	−22.45	−22.66	−21.11	−23.98	−22.37	−22.50	−26.07	−25.78	−28.88	−24.22	−23.56	−25.88	−25.20	−24.32	−24.82	−23.84	−22.01	−22.97
NLE, %	24.20	31.60	30.64	24.12	30.08	27.60	51.80	67.12	45.96	40.95	52.68	35.48	10.00	18.24	16.40	7.25	10.48	9.80

**Table 3:** The effect of ECH/α-CD molar ratios on the Z_ave_., ZP and PDI of α-CDNS.

ECH/αCD	6/1	8/1	10/1
Z_ave_. (nm)	442.40	313.10	262.80
PDI	0.546	0.409	0.481
ZP (mV)	−27.90	−33.60	−25.10

**Table 4:** The effect of ECH/α-CD molar ratios on the Z_ave._, ZP, PDI and NLE values of α-CDNS loaded with NAP.

ECH/αCD	6/1			8/1			10/1		
NAP/αCDNS	1/4	1/6	1/8	1/4	1/6	1/8	1/4	1/6	1/8
Z_ave_. (nm)	474	496	510	656.0	665.3	620.2	285	299	361
ZP (mV)	−24.50	−25.80	−24.56	−26.07	−25.78	−28.88	−21.25	−22.22	−23.44
PDI	0.575	0.699	0.652	0.537	0.418	0.575	0.486	0.443	0.525
NLE, %	20.80	25.56	21.68	51.80	67.12	45.96	22.20	26.40	20.28

**Table 5:** The effect of NaOH concentration on the Z_ave_., ZP and PDI of α-CDNSs.

NaOH (*w*/*w*)	25%	33%	40%
Z_ave_. (nm)	241.90	313.10	152.40
ZP (mV)	−27.93	−33.60	−24.20
PDI	0.423	0.409	0.479

**Table 6:** ZP, Z_ave._, and PDI values of α-CDNS synthesized at different NaOH concentration and their corresponding NLE values.

NaOH (*w*/*w*)	25%			33%			40%		
NAP/α-CDNS (*w*/*w*)	1/4	1/6	1/8	1/4	1/6	1/8	1/4	1/6	1/8
Z_ave_. (nm)	322.80	491.0	255.60	656.0	665.3	620.2	323.10	312.20	421.60
ZP (mV)	−24.20	−24.35	−23.59	−26.07	−25.78	−28.88	−22.82	−22.50	−22.89
PDI	0.711	0.457	0.407	0.537	0.418	0.575	0.474	0.503	0.510
NLE, %	7.44	14.80	18.88	51.80	67.12	45.96	23.72	31.60	34.56

## Data Availability

Data are contained within the article.
